# Inhibiting tumor necrosis factor-alpha diminishes desmoplasia and inflammation to overcome chemoresistance in pancreatic ductal adenocarcinoma

**DOI:** 10.18632/oncotarget.13212

**Published:** 2016-11-08

**Authors:** Xianda Zhao, Wei Fan, Zhigao Xu, Honglei Chen, Yuyu He, Gui Yang, Gang Yang, Hanning Hu, Shihui Tang, Ping Wang, Zheng Zhang, Peipei Xu, Mingxia Yu

**Affiliations:** ^1^ Department of Clinical Laboratory & Center for gene diagnosis, Zhongnan Hospital of Wuhan University, Wuhan, Hubei 430071, China; ^2^ Microbiology, Immunology and Cancer Biology Graduate Program, University of Minnesota, Minneapolis, Minnesota, 55455, USA; ^3^ Department of Pathology, Zhongnan Hospital of Wuhan University, Wuhan, Hubei, 430071, China; ^4^ Department of Pathology, School of Basic Medical Science, Wuhan University, Wuhan, Hubei, 430071, China; ^5^ Biomedical Sciences Graduate Program, Temple University, Philadelphia, Pennsylvania, 19140, USA

**Keywords:** pancreatic adenocarcinoma, tumor microenvironment, tumor necrosis factor, desmoplasia, chemoresistance

## Abstract

**Background:**

Pancreatic ductal adenocarcinoma (PDAC) is one of the most common cancer death reasons. Anti-tumor necrosis factor-alpha (TNF-α) antibodies have shown promising effects in PDAC pre-clinical models. However, the prognostic values of TNF-α, underlying mechanisms by which anti-TNF-α treatments inhibit PDAC, and potential synergistic effects of anti-TNF-α treatments with chemotherapy are still unclear.

**Results and Methods:**

To identify the targeting values of TNF-α in PDAC, we measured TNF-α expression in different stages of PDAC initiation and evaluated its prognostic significance in a pancreatic cancer cohort. We found that TNF-α expression elevated in PDAC initiation process, and high expression of TNF-α was an independent prognostic marker of poor survival. We further evaluated anti-tumor effects of anti-TNF-α treatments in PDAC. Anti-TNF-α treatments resulted in decreased cell viability in both PDAC tumor cells and pancreatic satellite cells in similar dose *in vitro*. *In vivo*, anti-TNF-α treatments showed effects in reducing desmoplasia and the tumor promoting inflammatory microenvironment in PDAC. Combination of anti-TNF-α treatments with chemotherapy partly overcame chemoresistance of PDAC tumor cells and prolonged the survival of PDAC mouse model.

**Conclusions:**

In conclusion, our findings indicated that TNF-α in PDAC can be a prognostic and therapeutic target. Inhibition of TNF-α synergized with chemotherapy in PDAC resulted in better pre-clinical responses via killing tumor cells as well as diminishing desmoplasia and inflammation in PDAC tumor stroma.

## INTRODUCTION

Pancreatic adenocarcinoma (PDAC) is a lethal disease, evidenced by the around five months' average survival time. [1, 2] The burden of PDAC was steadily increasing in past decades and is projected to be the second lethal cancer in 2030 in the United States. [3, 4] Due to the high aggressiveness of PDAC, only a small proportion of PDAC patients get the chance to receive surgical resection, which offers the only chance to cure. [1, 2] The gemcitabine-based conventional chemotherapy remains the major option for most PDAC patients, however it provides limited overall survival (OS)benefit. [5, 6]

Tumor necrosis factor-alpha (TNF-α) is a master regulator of inflammation and a key player in the cytokine network. [7, 8]. TNF-α is a type II transmembrane protein with signaling potential as a membrane-integrated protein or a soluble cytokine released by proteolytic cleavage. [9–11] There are two TNF receptors: TNFR1, which is activated by soluble ligand and TNFR2 which primarily binds the transmembrane form. Activation of TNFR1 and TNFR2 leads to various cellular processes including activation of apoptosis pathway, AP1 transcription factor and NF-κB pathway in different contexts. TNF-α was named for its ability to induce necrosis in some experimental cancers. [12] But further studies indicated that TNF-α has strong pro-cancer actions. Anti-TNF-α antibodies and other TNF-α antagonists showed therapeutic activity in various experimental models of common epithelial cancers. [13–17] Therefore, more studies are needed to clarify the functions of TNF-α in cancers.

The presence of heavy desmoplasia is a hallmark of PDAC forming a unique microenvironment that comprises various cell types including pancreatic stellate cells (PSCs), inflammatory and immune cells, endothelial cells and extracellular matrix (ECM). [18] During the progression of PDAC, large amount of PSCs and inflammatory cells were recruited into PDAC microenvironment. [18] Interactions between those stromal cells and tumor cells promote PDAC progression. [18–20] In cancers, TNF-α can be produced by tumor cells, stromal fibroblasts, and inflammatory cells. TNF-α in the tumor microenvironment further stimulates the production of other cytokines and chemokines. [7, 8] As the consequence, primary tumor growth and metastases, angiogenesis, and chemoresistance are enhanced, and the immune evasive tumor microenvironment is established. [7, 8]

The previous study showed that anti-TNF-α antibodies demonstrated promising anti-cancer effects in pre-clinical models of PDAC. [15] However, the clinical significance of TNF-α in PDAC, underlying anti-PDAC mechanisms, and potential chemotherapy synergistic effects of anti-TNF-α treatments were not investigated. In the current study, we sought to clarify these issues. We found that TNF-α expression was associated with PDAC initiation and predicted poor survival of PDAC patients. Inhibiting of TNF-α with anti-TNF-α antibodies reduced both tumor and stromal components, suppressed the inflammatory tumor microenvironment, resulting in synergistic effects with chemotherapy in a PDAC pre-clinical model.

## RESULTS

### Overexpression of TNF-α is associated with PDAC development and is on PDAC tumor cells and PSCs

**Figure 1:**
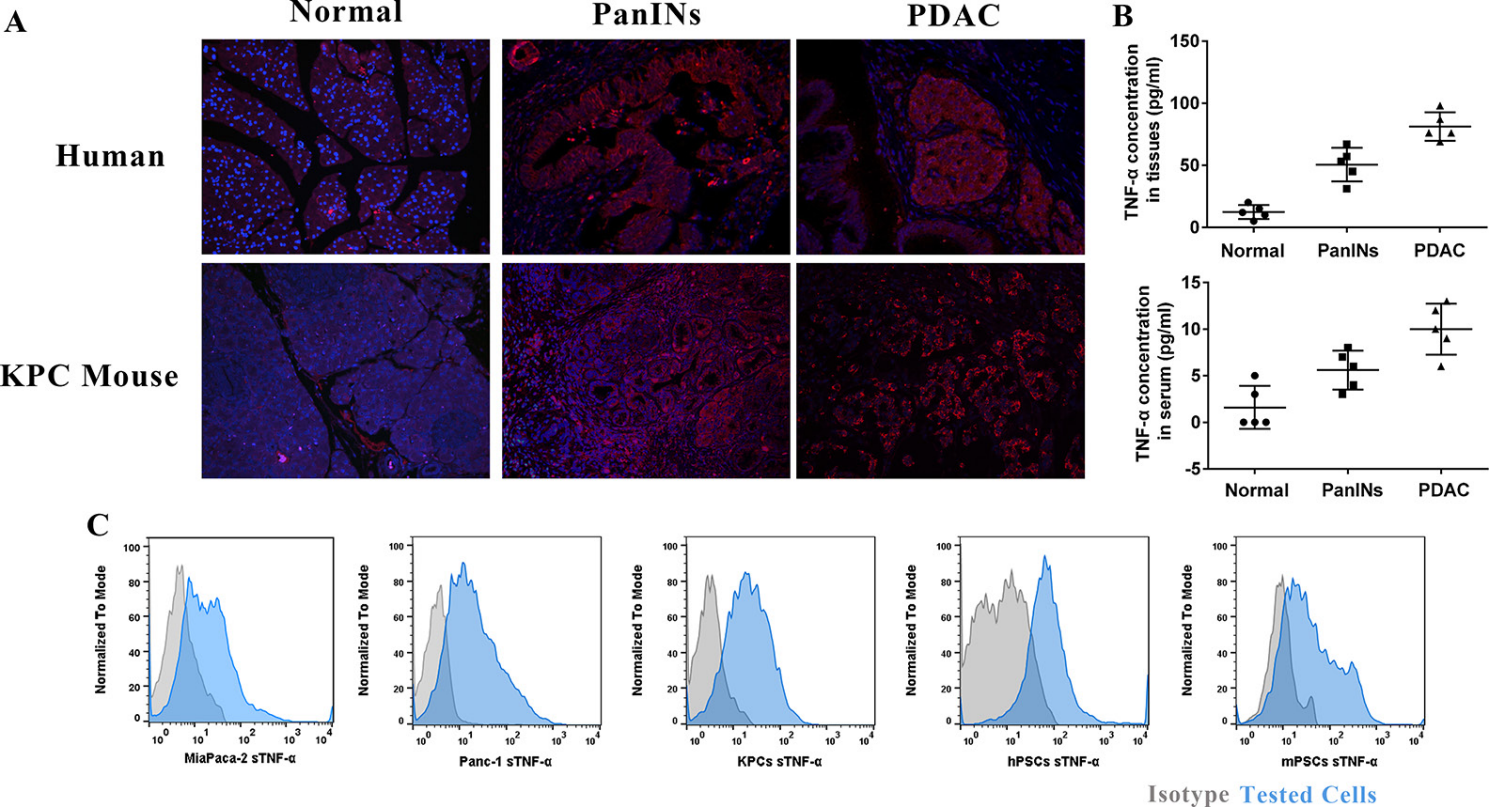
TNF-α overexpressed during PDAC initiation and in PDAC tumor and PSCs cell lines (**A**) Compare to normal pancreas, PanIN and PDAC showed higher expression of TNF-α. (**B**) Quantification of TNF-α in normal pancreas, PanIN, and PDAC lesions showed an increasing trend of TNF-α during PDAC initiation. The serum TNF-α showed same trend. (**C**) TNF-α highly expressed in both PDAC tumor cell lines (Panc-1, MiaPaca-2 and KPCs) and stromal PSCs cell lines (hPSCs and mPSCs).

To understand roles of TNF-α in PDAC initiation, we sought to determine whether TNF-α expression level is aberrant in pancreatic intraepithelial neoplasia (PanIN) lesions, PDAC lesions, PDAC tumor cells, and PSCs cell lines. We found that high expression of TNF-α was mainly shown in PanIN and PDAC lesions, but not in normal pancreas (Figure 1A). The histological features of these lesions were showed in Supplementary Figure S2. Quantification of TNF-α concentration in PDAC initiation indicated an increasing trend during PDAC development (Figure 1B). Additionally, expression of TNF-α was identified in PDAC cell lines Panc-1, MiaPaca-2, and KPCs as well as PSCs cell lines mPSCs and hPSCs (Figure 1C). These data, taken together, suggested that TNF-α is associated with PDAC initiation and is overexpressed in both PDAC lesions and surrounding PSCs.

### Expression of TNF-α is an independent prognosticator of PDAC patients

**Figure 2:**
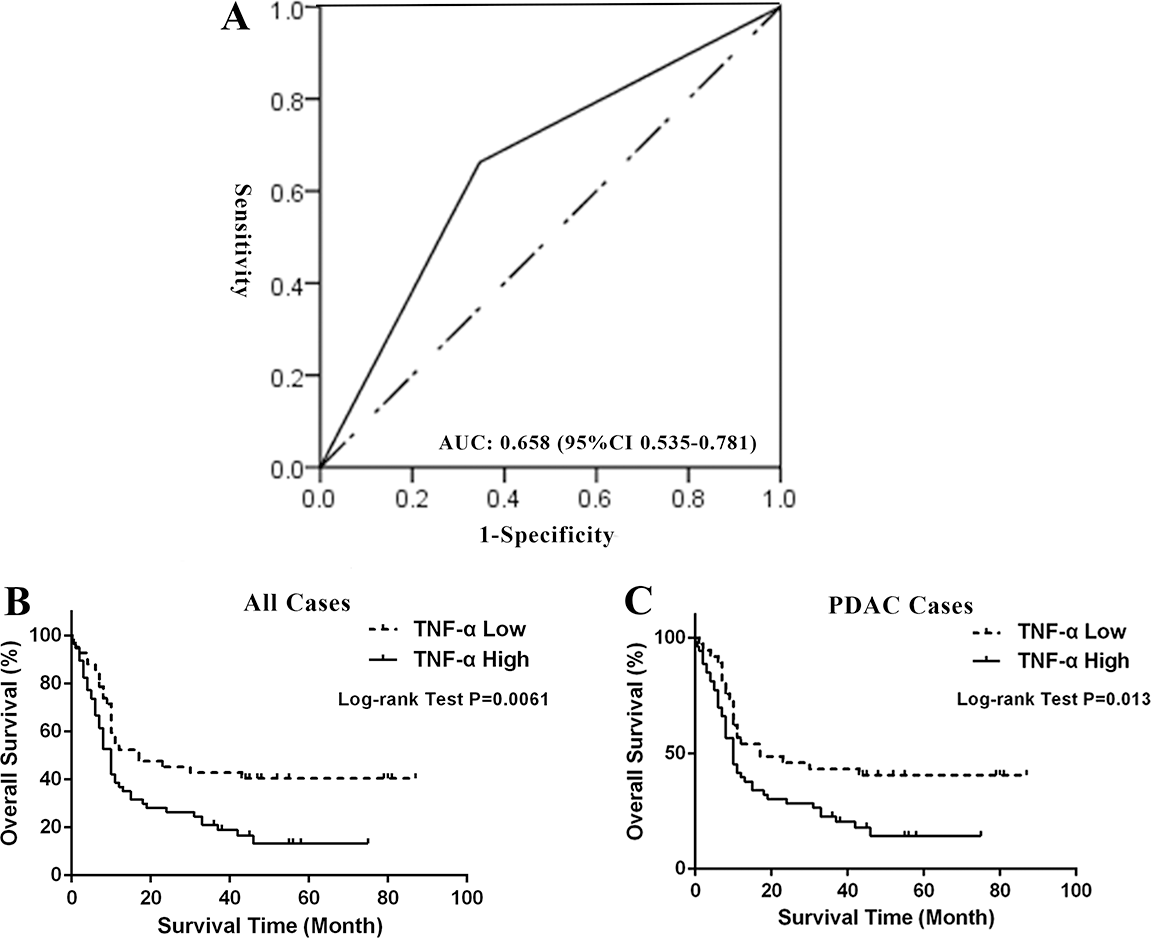
High expression of TNF-α predicted poor survival of PDAC patients (**A**) Cut-off point of low or high TNF-α expression was determined by ROC analysis. ID score of 4 is selected as the cut-off with largest area under curve (0.658 and 95% CI: 0.535 to 0.781, *P* = 0.017). (**B**) Kaplan-Meier curve and Log-rank test indicated high expression of TNF-α predicted poor OS of pancreatic cancer patients (*P* = 0.0061). (**C**) Kaplan-Meier curve and Log-rank test indicated high expression of TNF-α predicted poor OS of PDAC patients (*P* = 0.013).

Because our data showed that expression of TNF-α is associated with PDAC initiation, we sought to determine whether TNF-α expression level could serve as an independent prognostic marker of PDAC patients. To that end, the association between TNF-α expression and overall survival (OS) in pancreatic cancer was evaluated using a retrospective cohort including samples of 100 pancreatic cancer patients (Supplementary Table S1). Ninety-one cases are PDAC and nine are other pathological types of pancreatic cancer. The follow-up time is from 1 to 87 months. An intensity distribution (ID) score of 4 was used as the cut-off of high and low TNF-α expression based on the result of ROC analysis (AUC: 0.658, 95% CI: 0.535 to 0.782) (Figure 2A). Among all the 100 pancreatic cancer cases, 58 (58%) cases were TNF-α high expression and 42 (%) cases were low expression. The correlation between TNF-α expression level and classic clinicopathological parameters, such as age, gender and TNM stage was analyzed. However, no significant relationship was found (Supplementary Table S1).

Furthermore, we investigated the prognostic value of TNF-α expression in all pancreatic cancer cases and PDAC cases. Kaplan-Meier analysis and the log-rank test showed that high expression of TNF-α in both all pancreatic cancer cases and PDAC cases predicted poor survival (*P* = 0.0061 and 0.013, respectively). In all pancreatic cancer cases, median OS of high TNF-α expression subgroup was 10 months (95% CI, 7.96–12.04), while the low TNF-α expression subgroup had a median OS of 12 months (95% CI, 3.22–20.78). In univariate analysis, TNM stage, pathological grade, lymph node status, and TNF-α level were found to be significantly associated with the OS of all pancreatic cancer patients (*P* = 0.0029, 0.0088, 0.0021 and 0.0071, respectively; Table 1) as well as PDAC patients (*P* = 0.0081, 0.0199, 0.009 and 0.0141, respectively; Table 1). To determine whether TNF-α expression is an independent predictor of pancreatic patients' survival, a multivariate analysis was performed using COX proportional hazard regression model. TNM stage, pathological grade, and lymph node status were considered as potential confounding factors and were included in the multivariate model. Again, TNF-α independently and significantly predicted outcomes in all pancreatic cancer cases as well as PDAC cases (HR = 1.735, 95% CI: 1.046-2.877, *P* = 0.0327; HR = 1.868, 95% CI: 1.097–3.183, *P* = 0.0214, respectively; Table 1). Taken together, our data revealed that TNF-α expression is not only associated with PDAC initiation but also an independent prognosticator of PDAC patients, suggesting the critical values of targeting TNF-α in pre-clinical and clinical settings.

**Table 1:** COX proportional hazard models on overall survival of pancreatic cancer patients

Factors	Univariate Analysis		Multivariate Analysis	
	*P* value	HR (95% CI)	*P* value	HR (95% CI)
All Cases				
Sex (Male vs. Female)	0.539	1.162 (0.72–1.874)		
Age (≥ 62 vs. < 62)	0.224	1.329 (0.841–2.102)		
TNM stage (IIB–IV vs. I–IIA)	0.003	2.029 (1.274–3.233)	0.311	1.862 (0.559–6.199)
T stage (T3–T4 vs. T1–T2)	0.879	0.956 (0.533–1.715)		
Lymph node (Yes vs. No)	0.002	2.147 (1.321–3.491)	0.528	1.483 (0.436–5.042)
Metastasis (Yes vs. No)	0.433	1.757 (0.429–7.188)		
Pathological Type (Ductal Adenocarcinoma vs. Others)	0.221	0.614 (0.281–1.341)		
Grade of AC (Poorly vs. Well and moderately)	0.009	1.893 (1.174–3.05)	0.002	2.615 (1.512–4.523)
TNF-α Expression (High vs. Low)	0.007	1.948 (1.199–3.166)	0.033	1.735 (1.046–2.877)
PDAC Cases				
Sex (Male vs. Female)	0.747	1.085 (0.661–1.782)		
Age (≥ 62 vs. < 62)	0.27	1.312 (0.810–2.126)		
TNM stage (III–IV vs. I–II)	0.008	1.935 (1.187–3.155)	0.282	1.941 (0.579–6.505)
T stage (T3–T4 vs. T1–T2)	0.986	0.995 (0.55–1.799)		
Lymph node (Yes vs. No)	0.009	1.968 (1.184–3.27)	0.55	1.46 (0.422–5.046)
Metastasis (Yes vs. No)	0.394	1.849 (0.451–7.588)		
Grade of AC (Poorly vs. Well and moderately)	0.019	1.817 (1.099–3.003)	0.001	2.863 (1.579–5.19)
TNF-α Expression (High vs. Low)	0.014	1.902 (1.139–3.178)	0.021	1.868 (1.097–3.183)

### TNF-α is aberrantly expressed in highly chemoresistant PDAC cells

**Figure 3:**
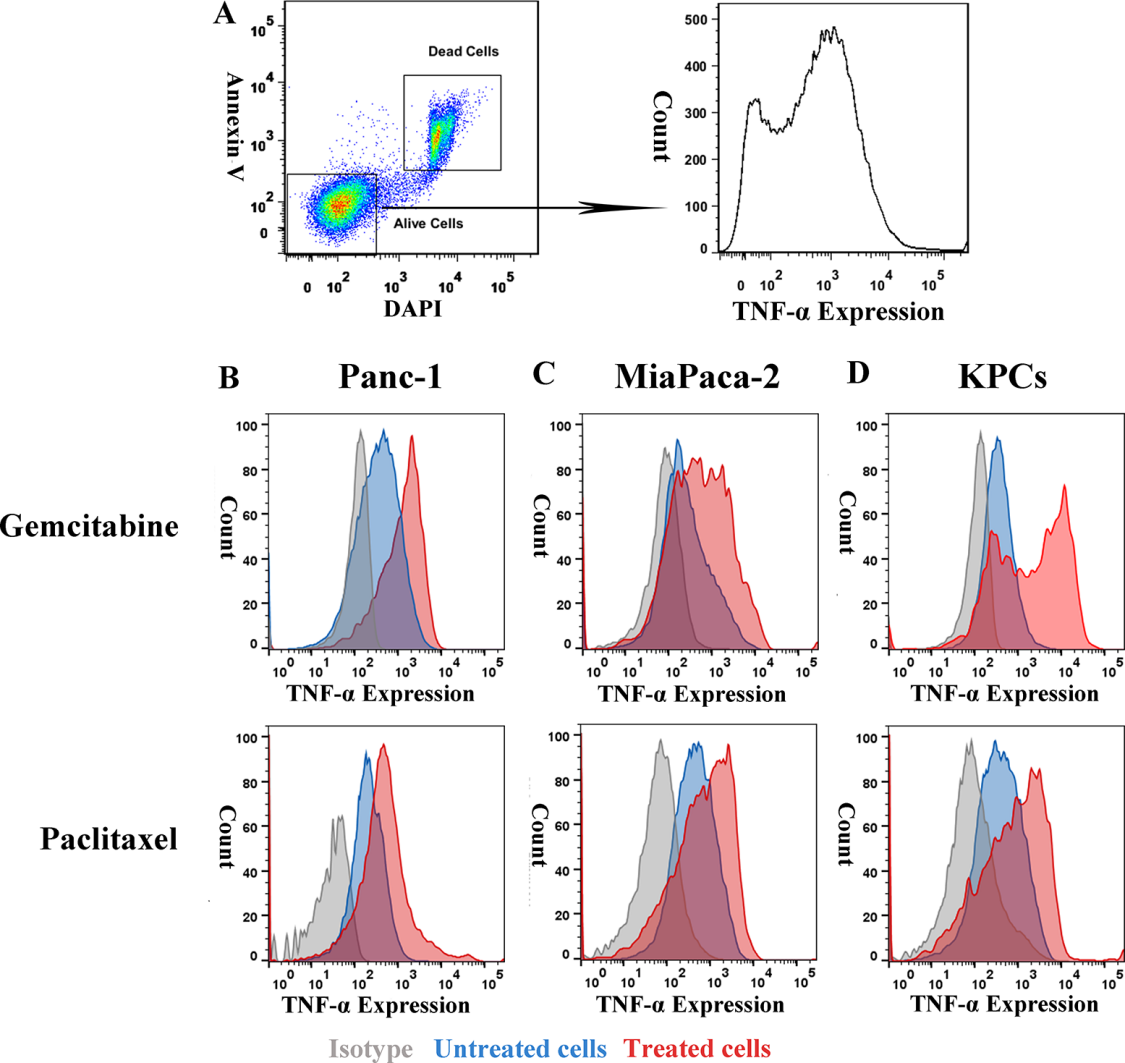
Expression of TNF-α was higher in PDAC tumor cells' chemoresistant subpopulation than general population (**A**) Gating strategy of chemoresistant PDAC cells. (**B**, **C** and **D**) Expression of TNF-α in normal and gemcitabine or paclitaxel resistant PDAC tumor cells. Compared with general PDAC cells population, gemcitabine, and paclitaxel resistant subpopulation showed higher TNF-α level.

To examine whether TNF-α expression level is associated with chemoresistance, we compared TNF-α expression between highly chemo-resistant PDAC cells and normal PDAC cells. Inhibitory concentration (IC50) dose was used as the treatment dose of each drug in cell lines (Supplementary Table S2). PDAC cell lines, Panc-1, MiaPaca-2 and KPCs were either untreated or treated with gemcitabine or paclitaxel for 48 h prior to FACS analysis. Cells were co-stained with Annexin V and DAPI to identify alive subpopulations that were further analyzed for TNF-α expression (Figure 3A). Expression of TNF-α was higher in chemo-resistant PDAC cells than untreated PDAC cells (Figure 3B–3D), suggesting that TNF-α involves in chemoresistance of PDAC tumor cells and is a potential target for overcoming the chemoresistance.

### Anti-TNF-α treatments inhibit viability of PDAC tumor cells via antibody-dependent cell-mediated cytotoxicity (ADCC) and complement-dependent cytotoxicity (CDC) effects and synergize with chemotherapy

The PDAC cell lines Panc-1, MiaPaca-2 and KPCs highly express TNF-α as showed in Figure 1C. To test if administration of anti-TNF-α treatments can kill PDAC cells, we performed direct cytotoxic assay, ADCC, and CDC assays. Without the presence of complement or immune effector cells, neither infliximab nor etanercept induced PDAC cells' death *in vitro* (data not shown). However, in the presence of complement or immune effector cells, both infliximab and etanercept reduced viability of PDAC tumor cells via inducing ADCC and CDC effects (Supplementary Figure S3). To test if anti-TNF-α treatment will synergize with chemotherapy to overcome chemoresistance, we combined infliximab with gemcitabine or paclitaxel in the presence of complement. Our data indicated that infliximab synergized with gemcitabine and paclitaxel in killing PDAC cells via CDC effects (Supplementary Figure S4). All these results demonstrated that PDAC cells are sensitive to anti-TNF-α treatments induced ADCC and CDC effects and combination of anti-TNF-α treatment with chemotherapy partially overcame PDAC chemoresistance *in vitro*.

### Anti-TNF-α treatments deplete PSCs *in vitro* and *in vivo*

**Figure 4:**
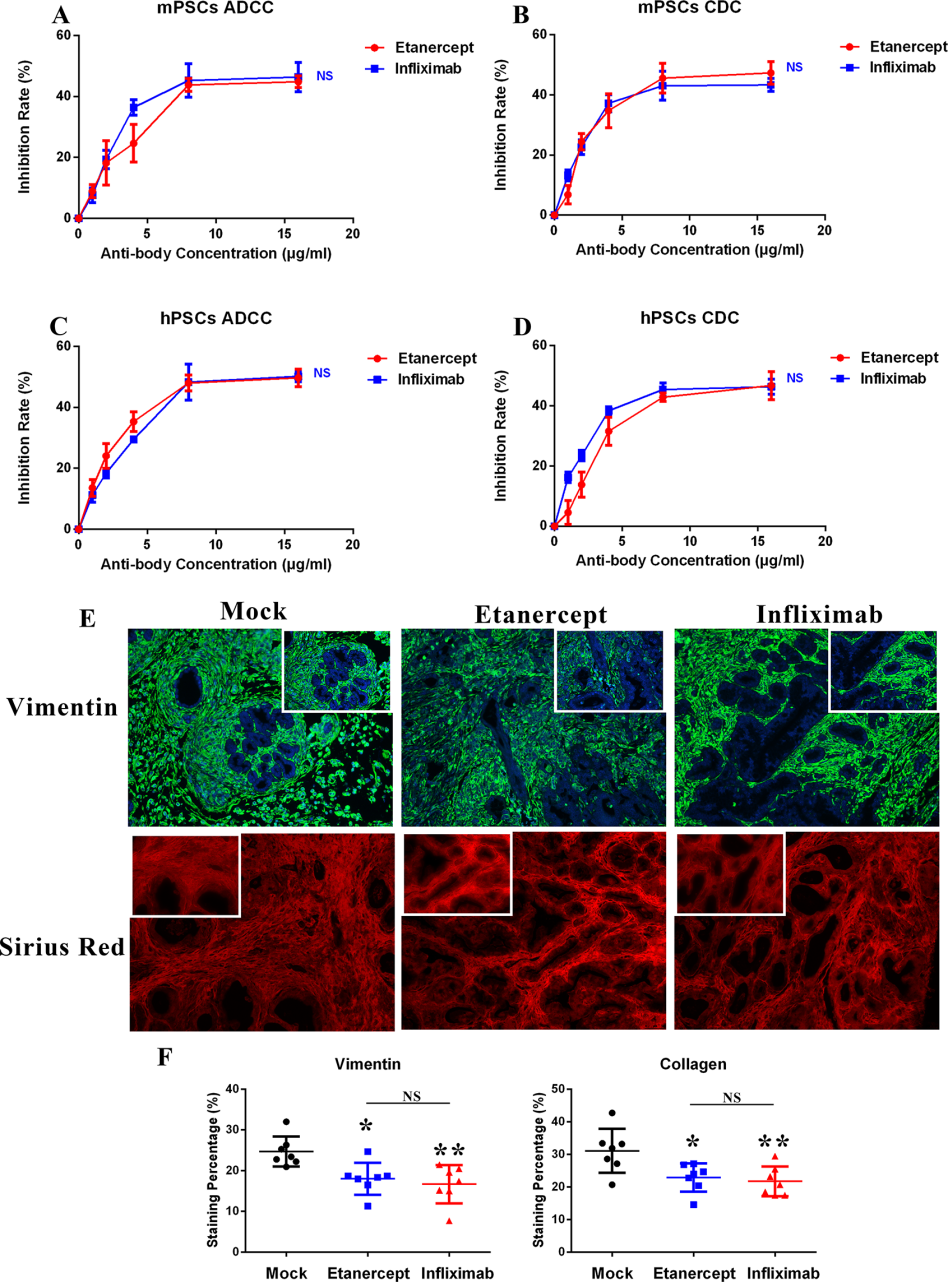
Anti-TNF-α treatments deplete desmoplastic elements *in vitro* and *in vivo* (**A**–**B**) Viability of mPSCs was inhibited by anti-TNF-α treatments induced ADCC and CDC effects. (**C**–**D**) Viability of hPSCs was inhibited by anti-TNF-α treatments induced ADCC and CDC effects. (**E**–**F**) In PDX model, anti-TNF-α treatments depleted PSCs cells (stained with vimentin) and collagen (stained with sirius red) deposition in PDAC tumor microenvironment (*n* = 7 in each treatment group). (*P < 0.05; **P < 0.01)

Due to our data indicated that TNF-α is highly expressed on two primary PSCs cell lines (Figure 1C), we sought to determine the effects of anti-TNF-α treatments on PSCs. To this end, we first evaluated the ADCC and CDC effects induced by anti-TNF-α treatment *in vitro*. As shown in Figure 4A–4D, viability of both mPSCs and hPSCs was inhibited by anti-TNF-α treatment in comparable rates with PDAC tumor cells (Supplementary Figure S3). In patient derived xenograft (PDX) model, our quantified data (Figure 4E and 4F) showed that anti-TNF-α treatments significantly decreased the number of PSCs, the major cellular component of PDAC stroma. In addition, the amount of collagen, main component of extra-cellular matrix was decreased by anti-TNF-α treatments as well. These findings demonstrated that the desmoplastic PDAC tumor stroma was obviously impaired by anti-TNF-α treatments.

### Anti-TNF-α treatments modulate inflammation in PDAC microenvironment

**Figure 5:**
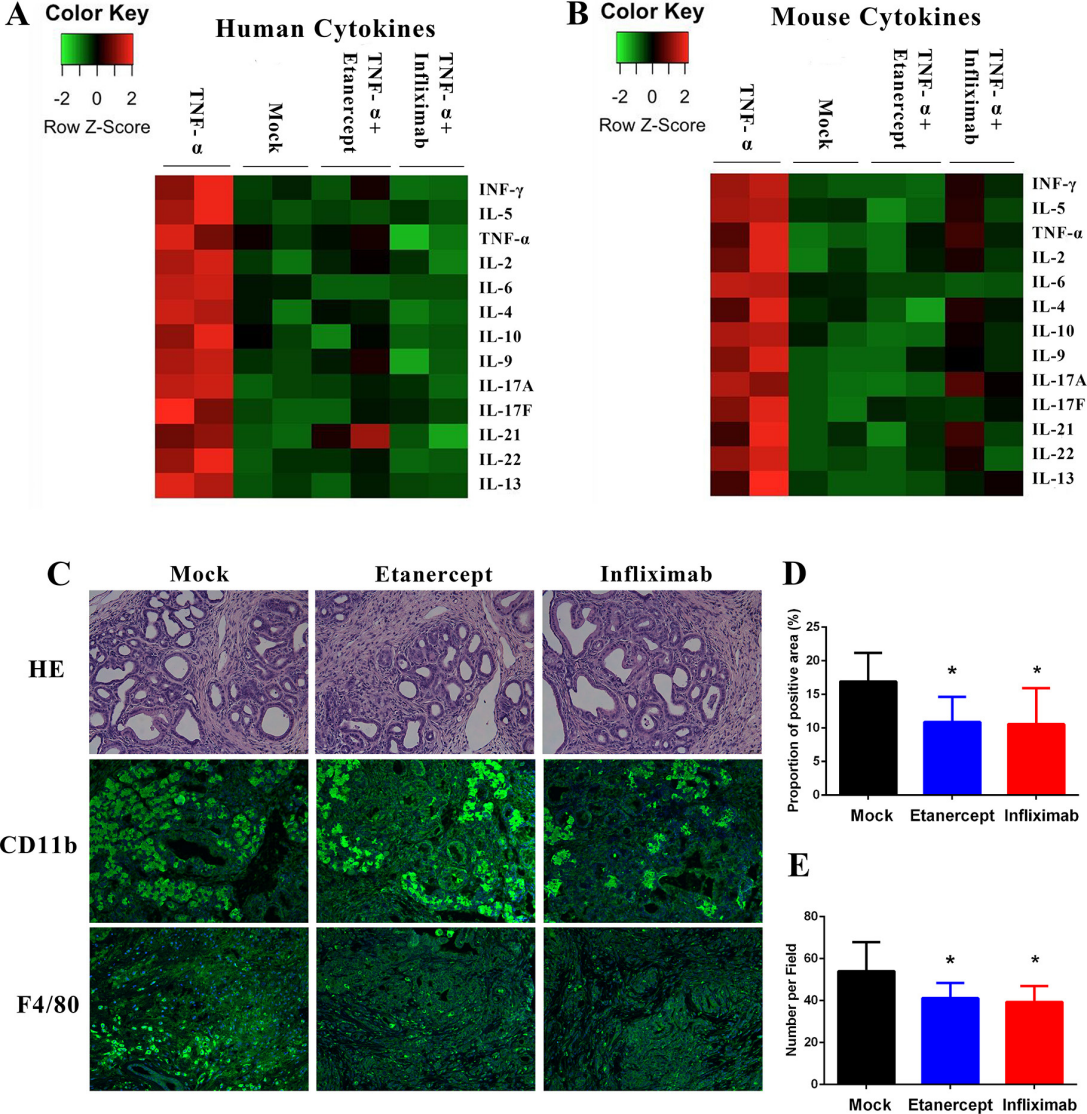
Anti-TNF-α treatments suppressed the inflammatory PDAC stroma (**A**–**B**) In PDX modes, exogenous TNF-α treatment induced expression of Th1 and Th2 cytokines, whereas anti-TNF-α treatments neutralized these effects. All evaluations were performed on PDX tumor tissue lysis. (**C**–**D**) Expression of CD11b was decreased by anti-TNF-α treatments in PDX model. (**C** and **E**) Number of F4/80 positive cells was decreased by anti-TNF-α treatments in PDX model. (*P < 0.05; **P < 0.01)

TNF-α in cancers is a master regulator of inflammation and the cytokine network. Here, we demonstrated that exogenous TNF-α administration obviously elevated the expression of mouse and human T helper cells related cytokines, such as INF-γ, IL-4, and IL-6 in tumors of PDX model (Figure 5A, 5B). When we administrated anti-TNF-α treatments, the cytokine production stimulating capacity of exogenous TNF-α in tumors of PDX model was neutralized (Figure 5A, 5B). Furthermore, we analyzed the inflammatory cellular components shifting after anti-TNF-α treatments. We found that number of CD11b+ and F4/80+ cells decreased after anti-TNF-α treatments in PDX model (Figure 5C–5E). These results, together with our findings that anti-TNF-α treatments depleted desmoplasia indicated the roles of anti-TNF-α in impairing the adverse tumor microenvironment of PDAC.

### Anti-TNF-α treatments synergize with chemotherapy *in vivo*

**Figure 6:**
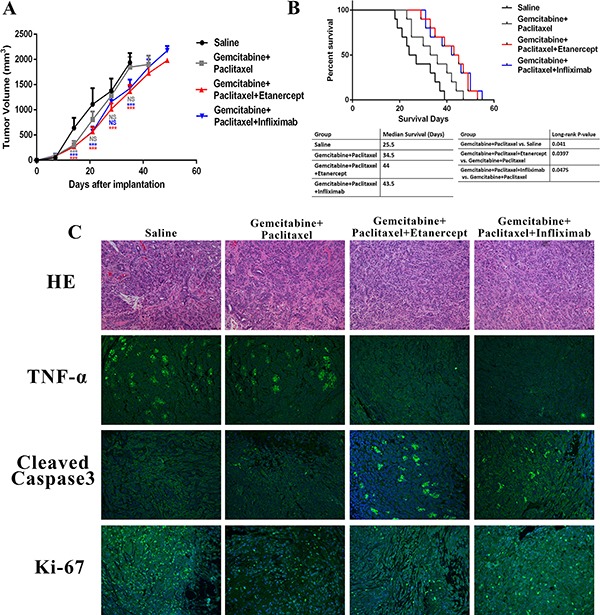
Combination of anti-TNF-α treatments with chemotherapy resulted in better anti-cancer responses in PDAC xenograft mouse model (**A**) Tumor growth curve showed that addition of anti-TNF-α antibodies further delayed tumor growth based on chemotherapy. (**B**) Anti-TNF-α treatments synergized with chemotherapy to prolong survival time of PDAC xenograft mouse model. (**C**) HE staining, TNF-α expression, cleaved-Caspase-3 expression, and Ki-67 expression were analyzed in each group. Less TNF-α expression and more cleaved-Caspase-3 expression were found in anti-TNF-α treatments groups.

Consider that anti-TNF-α treatments overcame chemoresistance vitro (Supplementary Figure S4), and reduced the tumor promoting tumor stroma, we further hypothesized that anti-TNF-α treatments will synergize with the standard chemotherapy in pre-clinical models of PDAC. To this end, we generated PDAC xenograft mouse model via injecting MiaPaca-2 cells subcutaneously into BALB/c nude mice. Mice were randomly split into four groups with different drug administrations: saline, gemcitabine plus paclitaxel, gemcitabine, paclitaxel plus infliximab, and gemcitabine, paclitaxel plus etanercept. Tumor volume was monitored. Our data revealed that in addition to tumor suppressive effects generated by gemcitabine and paclitaxel, infliximab and etanercept further delayed tumor growth (Figure 6A). Survival time is a strong indicator of therapeutic benefits. Compared with saline and chemotherapy group, combination of chemotherapy with anti-TNF-α treatment showed around 20 and 10 days' longer survival time, respectively (Figure 6B). Log-rank test showed statistical significance between chemotherapy along and chemotherapy pluses anti-TNF-α treatment groups(Figure 6B).

Our further mechanistic studies revealed that the amount of cleaved-Caspase-3 was higher in combination treatment group than chemotherapy and saline groups (Figure 6C, Supplementary Figure S5A). Besides tumor cell apoptosis, we also checked if the combination treatment could also inhibit tumor cell proliferation to delay tumor growth. However, combination of anti-TNF-α treatment didn't enhance the proliferation inhibition role of chemotherapy (Figure 6C, Supplementary Figure S5B). In summary, anti-TNF-α treatment synergized with standard chemotherapy in PDAC mouse model to delay tumor growth and prolong mice survival via enhancing tumor apoptosis.

## DISCUSSION

Despite the improvements in our understanding of PDAC, it remains a clinical challenge and a major cause of cancer mortality. [1, 2] Surgical resection still offers the only chance to cure. However, only around 15% PDAC patients can benefit. Gemcitabine based combinational chemotherapy chemoresistance is the standard therapies for most PDAC patients. [5, 6] However, efficacy of chemotherapy is limited by the rapidly generated adaptive chemoresistance. Novel therapeutic strategies that synergize with current standard care are therefore desperately needed. Anti-TNF-α antibodies have shown therapeutic benefits on various pre-clinical cancer models including orthotopic PDAC pre-clinical model. [13–17] However, the clinical and targeting values of TNF-α in PDAC, chemotherapy synergistic effects, and underlying anti-PDAC mechanisms of anti-TNF-α treatments remain unclear.

To clarify the targeting and prognostic values of TNF-α in PDAC, we investigated TNF-α expression in different stages of PDAC initiation and correlated it to clinical parameters and prognosis of PDAC patients. Our results demonstrated that high transmembrane and total TNF-α level was associated with PDAC initiation. Transmembrane TNF-α also overexpressed in PDAC cell lines and PSCs isolated from full PDAC tumors. Our further analysis of TNF-α expression in pancreatic cancer patients indicated that high TNF-α expression predicted poor survival. Results of multivariate regression model further strengthen the prognostic values of TNF-α expression, as it was identified as an independent prognostic marker of pancreatic cancer. Taken together, our data suggested that TNF-α in PDAC is tumor-promoting, a maker of survival prediction, and more importantly a potential target for treatment.

The previous study reported that transmembrane TNF-α was highly expressed in chemoresistant leukemia cells, and inhibiting transmembrane TNF-α reversed chemoresistance. [13] In line with this report, we found that transmembrane TNF-α expression was up-regulated in gemcitabine and paclitaxel treated PDAC cells when compared to untreated PDAC cells. In the next step, we sought to understand if the combination of anti-TNF-α treatments will synergize with chemotherapy in killing PDAC cells. Two distinct types of anti-TNF-α drugs: infliximab, a humanized TNF-α monoclonal antibody, as well as etanercept, a TNF receptor to the constant end of the IgG1 antibody were administrated. Previous research reported these two drugs could induce cell death via ADCC and CDC effects. [21] In our study, we found that these two anti-TNF-α drugs significantly reduced PDAC cells' viability via inducing ADCC and CDC effects. Furthermore, by the CDC effect, anti-TNF-α treatment partially overcame the chemoresistance of PDAC cells to gemcitabine and paclitaxel *in vitro*. *In vivo* studies using xenograft PDAC mouse model recapitulated the chemotherapy synergistic roles of anti-TNF-α treatments. In summary, it is therefore interesting to note that combining anti-TNF-α treatments might be an option in overcoming chemoresistance and prolong survival time in PDAC patients.

Tumors are a heterogeneous milieu of epithelial cells, stromal cells and non-cellular constituents. [18, 20] In pancreatic cancer, the heavy desmoplasia is considered to be the major challenge of conventional and targeted therapy. [18, 22, 23] It was known that PSCs and the ECM produced by PSCs that associated with tumor fibrosis are tumor promoting in PDAC. [19, 23] Drugs that target tumor stroma specifically or tumor and stoma simultaneously were promisingly tested in pre-clinical animal models. [24–26] As TNF-α also overexpressed in PSCs isolated from PDAC, we hypothesized that anti-TNF-α treatments will show anti-desmoplasia effects on PDAC. Interestingly, anti-TNF-α treatments effectively inhibited PSCs' viability via ADCC and CDC effects *in vitro*. In PDX model, anti-TNF-α antibodies depleted desmoplasia via reducing PSCs population and ECM production, suggesting a novel mechanism by which anti-TNF-α treatments inhibit PDAC development.

There are accumulating evidences that chronic inflammation promotes tumorigenesis, favors angiogenesis and enhances tumor spreads. [27, 28] In many inflammatory scenarios, TNF-α plays a central role. [8] We therefore investigated roles of TNF-α and anti-TNF-α treatment in modulating the inflammatory PDAC tumor microenvironment. Compared with control, infusion of TNF-α stimulated the inflammatory tumor microenvironment of PDAC. However, these inflammatory stimulating effects were well neutralized by anti-TNF-α treatments. It need to be noted that anti-TNF-α agents have various affinity to different types of TNF-α. In our experiment, we didn't see significant discrepancy between infliximab and etanercept in decreasing the inflammatory cytokines in animal model. This may be because the amount of infused anti-TNF-α agents was high enough to cover any difference cause by various reactive affinity. These findings are critical as they indicated that inhibiting the tumor-promoting inflammation could also be a possible mechanism of anti-TNF-α treatments induced PDAC regression. In summary, the present study confirmed the targetingvalue of TNF-α in PDAC, and revealed the mechanisms by which anti-TNF-α treatments induce PDAC regression. More importantly, our study demonstrated the synergistic roles of anti-TNF-α treatments with chemotherapy in PDAC, suggesting a promising novel therapeutic option for PDAC patients.

## MATERIALS AND METHODS

### Cell lines and cell culture

Human PDAC cell lines, Panc-1 and MiaPaca-2 were purchased from American Type Culture Collection and cultured in Dulbecco's Modified Eagle's Medium (DMEM, Gibco, ThermoFisher Scientific, NY, USA) with 10% fetal bovine serum (FBS) and 1% Pen-strap (Giboco). Primary cell lines hPSCs and mPSCs were isolated from surgical resected human PDAC tissues and KrasLSL.G12D/+; p53R172H/+; PdxCretg/+ (KPC) mice PDAC tumor tissues respectively by following procedure described by Sharon et al. [29] hPSCs and mPSCs were cultured in Iscove's Modified Dulbecco's Medium (IMDM, Gibco) with 10% FBS and 1% Pen-strap (Giboco). Primary cell line KPCs was isolated from KPC mice tumor tissues with the procedure reported by Johannes et al. [30] and cultured in DMEM plus 10% FBS and 1% Pen-strap (Giboco). Purity of primary cell lines were checked by both morphology and molecular markers (Supplementary Figure S1).

### Cell viability assay

Cell viability was determined using Dojindo Cell Counting Kit-8 (CCK8). After treatment with cytotoxic drugs in different concentrations, 10 μl of CCK-8 was added for 1 h incubation at 37°C. Then absorbance at 450nm was measured. Cell viability was calculated by: (OD450nm (Treatment)-OD450nm (Blank))/(OD450nm (Control)-OD450nm (Blank)).

### Antibody-dependent cell-mediated cytotoxicity and complement-dependent cytotoxicity assays

Two Food and Drug Administration approved drugs infliximab and etanercept were used for anti-TNF-α purposes in current study. Both of these two drugs were able to react with mouse TNF-α and were used in previous studies. [31, 32] For ADCC assay, peritoneal macrophages from C57BL/6 mice served as effector cells for mouse derived target cells, and human peripheral blood mononuclear cells were used as effector cells for human target cells. All PDAC and PSCs cell lines in this study were used as target cells. Before mixing the effector cells and target cells (2,000 cells/well) at a ratio of 20:1, target cells were pre-incubated with infliximab or etanercept for 30 minutes. For CDC assay, targets cells (2,000 cells/well) exposed to 5% fresh guinea pig serum with active complement and infliximab or etanercept for 4 hours' incubation at 37°C. Cell viability of ADCC and CDC assays was measured by CCK-8 method as described above. Cytotoxicity of ADCC and CDC were calculated as previous publication by Yu et al. [14].

### Fluorescence activated cell sorter analysis

Expression of TNF-α was detected with fluorescence activated cell sorter analysis (FACS, FACSCalibur 440E, Becton Dickinson, USA). Cells were collected from fresh cell culture, washed and suspended to single cell suspension. Primary antibody TNF-α (ab1793, Abcam, CA, USA) was incubated on ice for 1 hour followed by washing and fluorescence labeled secondary antibody for 15 minutes at room temperature. Apoptosis analysis by FACS was conducted using DAPI and Annexin-V (BD Biosciences, CA, USA) according to the manufacturer's instructions. Only transmembrane TNF-α was measured as cells were not permeabilized before TNF-α staining. Data were visualized by FlowJo software.

### Animal models

All animal studies were approved by the Animal Care and Use Committee of Wuhan University. Six week old female BALB/c nude mice (18–22 g; Shanghai SLAC Laboratory Animal Co., Ltd.) were housed in specific pathogen-free environment with a 12-hour light-dark cycle. A total of 5 × 10^6^ MiaPaca-2 cells were inoculated subcutaneously into the flanks of hind legs. Seven days after inoculation, mice were randomized into four groups (10 mice/group): saline, gemcitabine (100 mg/kg/week) + paclitaxel (10 mg/kg/week), gemcitabine (100 mg/kg/week) + paclitaxel (10 mg/kg/week) + etanercept (5 mg/kg/3days) and gemcitabine (100 mg/kg/week) + paclitaxel (10 mg/kg/week) + infliximab (10 mg/kg/week). Treatment started from day 8 after inoculation and tumors were monitored every 7 days with microcalipers. Tumor volume was calculated by length × width^2^ × π/6. For the survival study, excessive ascites, more than 20% body weight loss or other signs of distress were considered as end point.

To evaluate expression of TNF-α during PDAC development, KPC mice (originated from Dr. Tuveson lab), constructed based on previous report. [33] were used. To study the effects of anti-TNF-α treatments on PDAC tumor microenvironment, orthotopic patient derived xenograft (PDX) mouse model was established. Resected human pancreatic tumors were implanted subcutaneously into female BALB/c nude mice. When tumor volumes reached 600 mm^3^, tumors were dissected and cut into 10 mm^3^ pieces by biopsy instrument. Tumor chunks were then implanted into pancreas of additional BALB/c nude mice. Animals were randomized and tagged prior to treatment (3 groups, *n* = 7/group). Treatment with saline, recombinant human TNF-α protein (R&D Systems, MN, USA, 0.5 mg/kg, single dose. This product also reacts with mouse cells), etanercept (5 mg/kg/3days), and infliximab (10 mg/kg/week) started 7 days after implantation. Animals were treated for 28 days prior to tissue collection.

### Cytokine analysis assay

The cytokine quantification analysis was performed by beads based assay. Human and mouse T helper cytokine panel (IL-2, IL-4, IL-5, IL-6, IL-9, IL-10, IL-13, IL-17A, IL-17F, IL-21, IL-22, IFN-γ and TNF-α) was purchased from Biolegend (CA, USA). PDX mice were treated with saline, TNF-α (10 μg/mouse/week), TNF-α (10 μg/mouse/week) + etanercept (5 mg/kg/3days) or TNF-α (10 μg/mouse/week) + infliximab (10 mg/kg/week). Two mice were in each group. After 28 days' treatment, tumors were resected and cut into pieces. Small tissue pieces were then minced and filtered to get cell precipitation. Proteins were extracted by RIPA buffer with protease inhibitor and quantified by BCA protein assay. Protein concentrations from different samples were normalized to the same before measurement. For the serum tests, same volume was taken from each sample. Remaining steps were performed according to the manufacturer's recommendations. Each sample was measured in duplicate and mean values were used in analysis.

### Histology analyses

For histology analyses, 4 μm paraffin embedded tissue sections were deparaffinized in xylene and hydrated through gradient ethanol. Hematoxylin and eosin (H&E) staining were performed to confirm histological features. To quantify collagen amount, tissue sections were stained using picrosirius red staining solution (Chondrex Inc, WA, USA) and imaged with Texas Red filter.

### Clinical sample analysis

Using of human specimens in this study was approved by the Medical Ethics Committee of Wuhan University. All PDAC specimens were obtained from PDAC patients by surgery with informed consent. Tissue microarrays containing a total of 100 patients' specimens were constructed by Shanghai Outdo Biotech (Shanghai, China). All these 100 patients were treated with Whipple procedure or cytoreductive surgery of PDAC, prior to administration of chemotherapy or radiotherapy. Histological diagnosis and grade of differentiation were reconfirmed by two board certified pathologists (Z.X. and H.C.) before staining. All of the PDAC samples were classified based on the AJCC TNM classification (7th Edition). Clinicopathological factors of PDAC patients were listed in Supplementary Table S1. Follow-up began on the date of surgery and ended in October 2012. OS of each patient was documented.

### Immunostaining and slides grading

Slides were first deparaffinized and hydrated, then were steamed with Reveal Decloaker (Biocare Medical, CA, USA) for 30 minutes for antigen retrieval. To minimize background staining, slides were incubated with 5% BSA buffer for 20 minutes at room temperature. Primary antibodies for TNF-α (Abcam), Vimentin (Abcam), cleaved-Caspase3 (Abcam), Ki67 (Abcam), CD11b (Abcam) and F4/80 (Abcam) were diluted according to vendor's instruction and were incubated overnight at 4°C. Alexa Fluor^®^ conjugated secondary antibodies were used after primary antibody staining. Slides were counterstained with mounting DAPI media.

To correlate TNF-α expression with clinical significances of PDAC, PDAC tissue microarrays were stained by immunohistochemistry. Slides were graded by two pathologists (Z.X. and H.C.) simultaneously in a same displayer. Both the two pathologists were independent and blinded to the clinical features of patients. The scores of the two pathologists were compared and any discrepant scores were reassessed by reevaluation of the stained tissue specimen to achieve a consensus score. The area of positive (AP) were graded as follows: 0 (no positive area or positive area < 5%), 1 (positive area 5–25%), 2 (positive area 26–50%), 3 (positive area > 51%). Intensity of staining (IS) by the numerical value was resulted in scores: 0 (negative: no positive signal), 1 (weak signal) 2 (medium signal) and 3 (strong signal). Expression levels of TNF-α were calculated based on the total score as the following equation: ID = AP × IS. The ID cutoff value for high and low expression was determined by the receiver operating characteristic (ROC) curve analysis with the respect to OS.

### Statistical analysis

Values were presented as mean ± SD. Two group quantification data were analyzed using *t*-test. Multigroup data were analyzed by one-way ANOVA following Bonferroni's pairwise comparisons. ROC curve analysis was conducted to determine the cutoff of ID and evaluate the predictive value of TNF-α expression for OS of pancreatic cancer patients. To analyze the correlation between the clinicopathological parameters and TNF-α expression, the χ^2^ test or Fisher's exact test was used. The OS was estimated using the Kaplan Meier method and was compared using the long-rank test. Multivariate analysis using Cox proportional hazard regression model was performed to test independent prognosis values. A two tailed *p*-value < 0.05 was considered statistically significant.

## SUPPLEMENTARY MATERIALS


