# Supplementary material for: Inhibiting tumor necrosis factor-alpha diminishes desmoplasia and inflammation to overcome chemoresistance in pancreatic ductal adenocarcinoma
Source: Oncotarget. 2016 Nov 8;7(49):81110–22. doi: 10.18632/oncotarget.13212 (PMC5348380; doi:10.18632/oncotarget.13212)
Supplement: Supplementary file 1 [file oncotarget-07-81110-s001.pdf]

Inhibiting tumor necrosis factor-alpha diminishes desmoplasia and inflammation to overcome chemoresistance in pancreatic ductal adenocarcinoma

Supplementary Materials

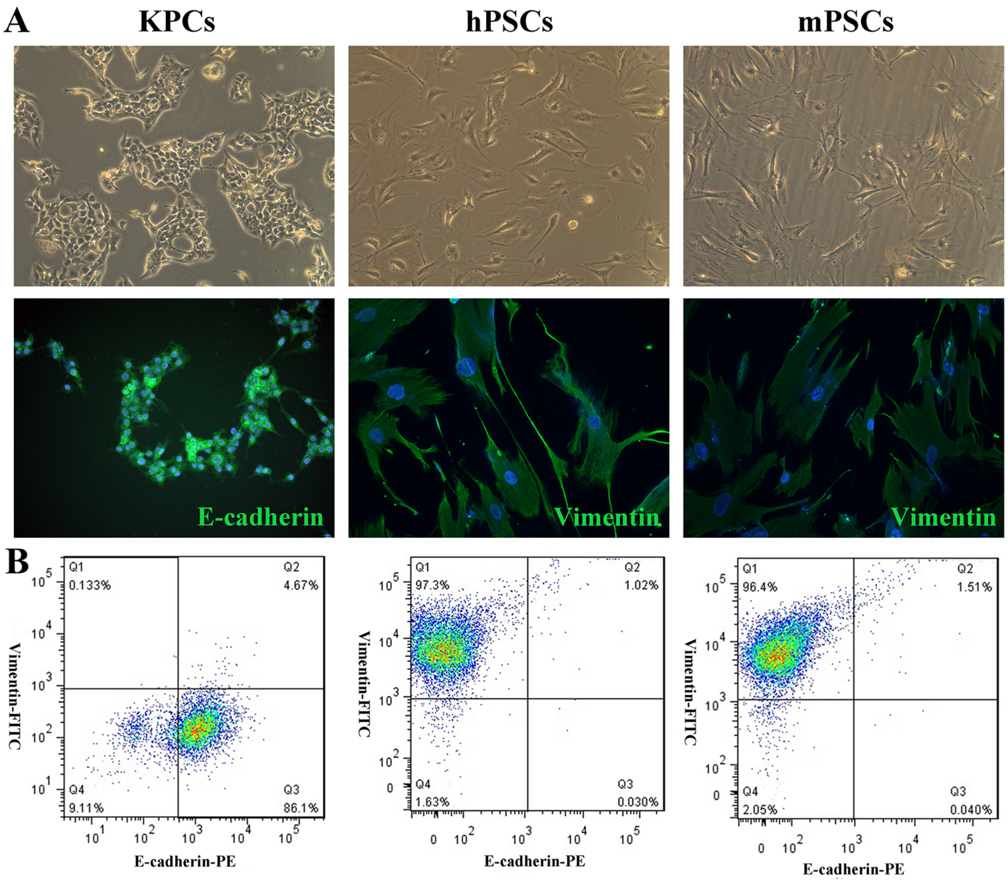

Supplementary Figure S1: Primary cell lines were identified by morphology and molecular markers. (**A**) Morphology of primary cell lines KPCs, hPSCs, and mPSCs. KPCs was stained by epithelia cell marker E-cadherin. hPSCs and mPSCs were stained by fibroblasts marker vimentin. (**B**) KPCs, hPSCs, and mPSCs were stained by both E-cadherin and vimentin. FACS analysis showed KPCs were E-cadherin positive only while hPSCs and mPSCs were vimentin positive only.

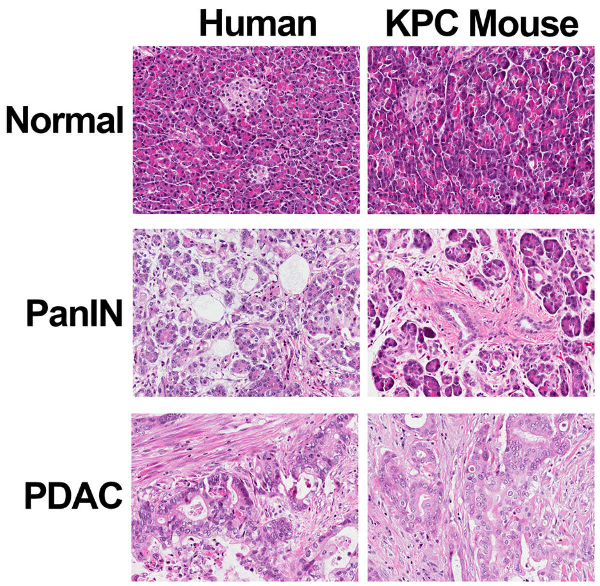

Supplementary Figure S2: HE staining of different pancreatic lesions. The HE staining of both human and KPC mouse normal, PanIN, and PDAC tissues were performed to show histological features.

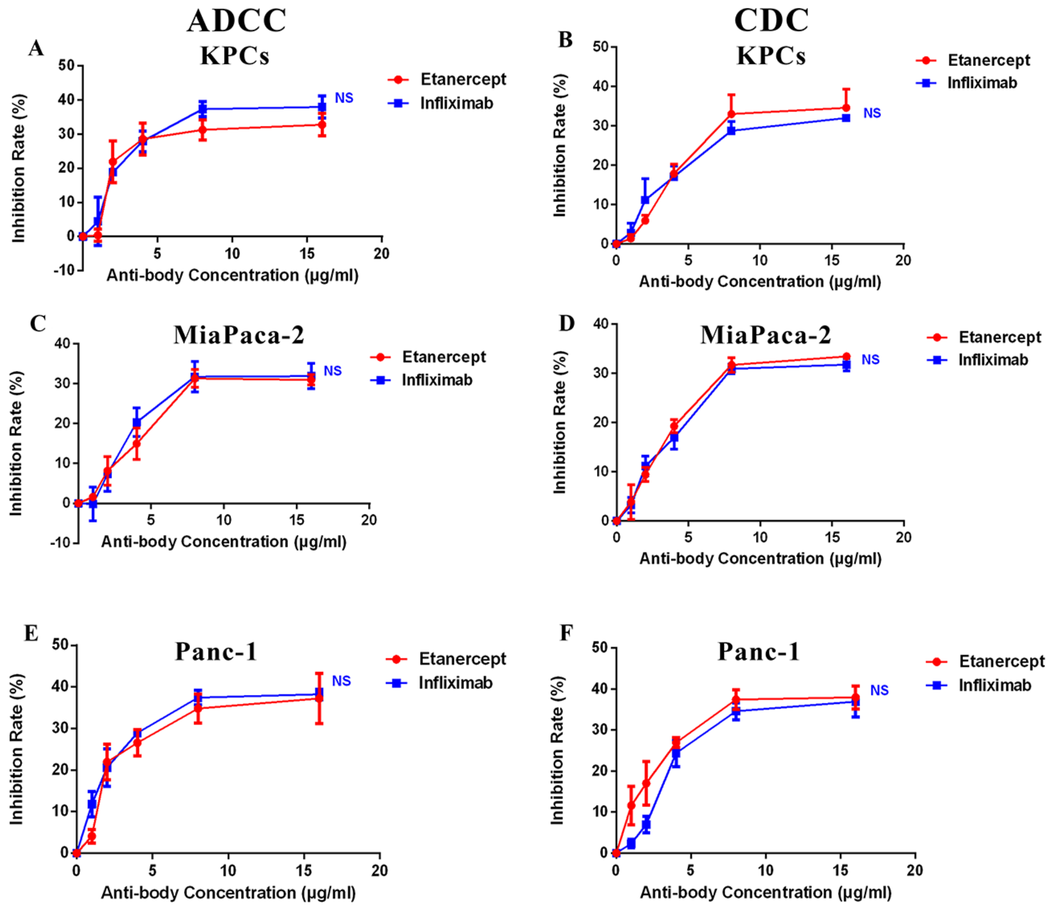

Supplementary Figure S3: Anti-TNF-α treatments induced ADCC and CDC effects to inhibit PDAC tumor cells' viability. (**A**–**B**) Cell viability of KPCs was reduced by ADCC and CDC effects induced by anti-TNF-α treatments. (**C**–**D**) Cell viability of MiaPaca-2 was reduced by ADCC and CDC effects induced by anti-TNF-α treatments. (**E**–**F**) Cell viability of Panc-1 was reduced by ADCC and CDC effects induced by anti-TNF-α treatments.

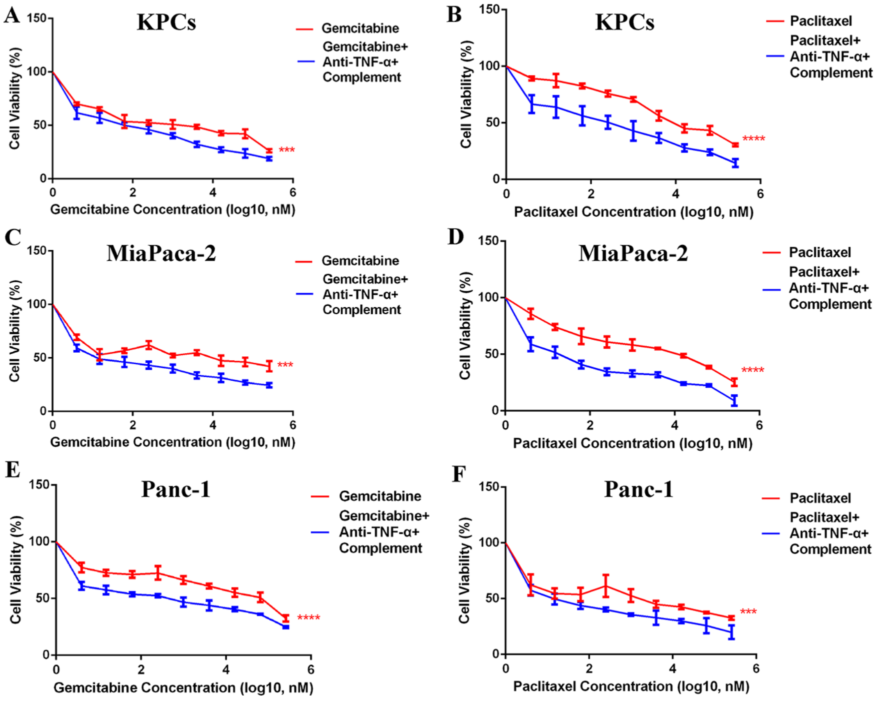

Supplementary Figure S4: Anti-TNF-α treatments partially overcame chemoresistance of PDAC tumor cells. (**A**–**B**) Anti-TNF-α treatment overcame KPCs gemcitabine and paclitaxel resistance via CDC effects. (**C**–**D**) Anti-TNF-α treatment overcame MiaPaca-2 cells gemcitabine and paclitaxel resistance via CDC effects. (**E**–**F**) Anti-TNF-α treatment overcame Panc-1 cells gemcitabine and paclitaxel resistance via CDC effects. (*$P < 0.05$; **$P < 0.01$)

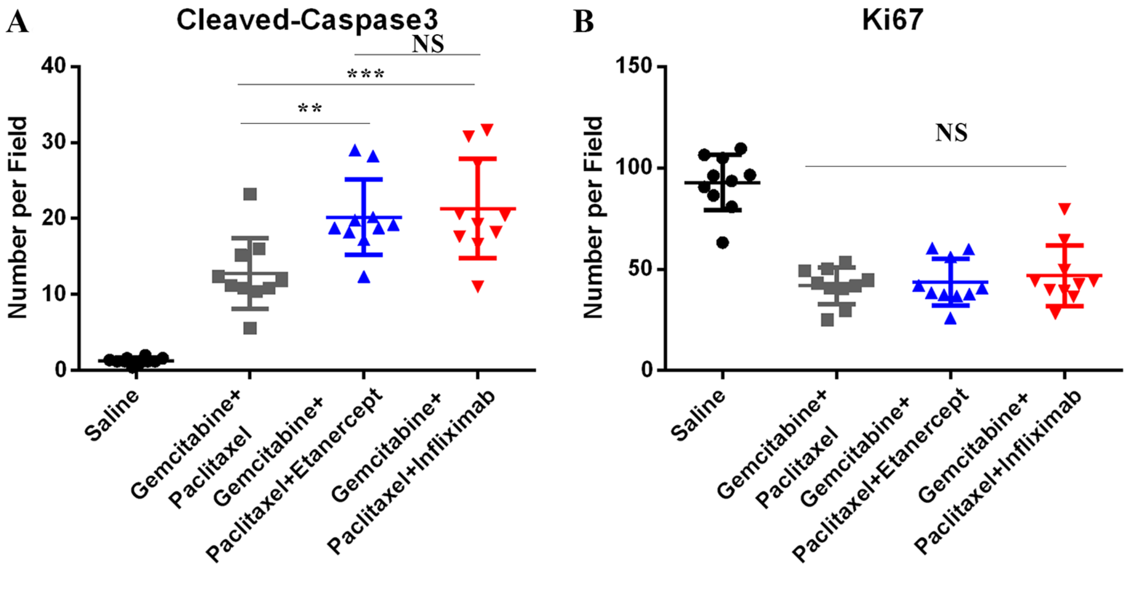

Supplementary Figure S5: Quantification of cleaved-Caspase-3 and Ki-67 expression in xenograft PDAC mouse model. (**A**) Compare with chemotherapy alone, combination of anti-TNF-α treatments with chemotherapy increased number of cleaved-Caspase-3 positive cells in xenograft tumors. (**B**) Combination of anti-TNF-α treatments with chemotherapy didn't further inhibit cell proliferation compared with chemotherapy alone in xenograft tumors. (*$P < 0.05$; **$P < 0.01$)

Supplementary Table S1: Correlations between TNF-α expression and clinicopathological parameters of pancreatic cancer

Parameters	*N*	TNF-α Expression		*P*-value
		Low (%)	High (%)	
**Age**				
< 62	56	27	29	0.155
≥ 62	44	15	29	
**Gender**				
Male	63	23	40	0.147
Female	37	19	18	
**T Stage**				
T1 + T2	52	26	26	0.109
T3 + T4	47	16	31	
**Lymph node status**				
Negative	54	27	27	0.176
Positive	39	14	25	
**Metastasis**				
Negative	98	41	57	0.817
Positive	2	1	1	
**TNM Stage**				
I + II	78	35	43	0.229
III + IV	20	6	14	
**Pathological Grade**				
Low and Medium	68	32	36	0.135
High	32	10	22	
**Pathological Type**				
Ductal Adenocarcinoma	91	37	54	0.388
Others	9	5	4	
**Chronic Pancreatitis**				
Yes	31	12	19	0.827
No	69	30	39	
**Survival Time**				
≤ 10 months	51	17	34	0.073
> 10 months	49	25	24	

Supplementary Table S2: IC50 dose of different PDAC cell lines

Cell line	Gemcitabine IC50 (nM)	Paclitaxel IC50 (nM)
KPCs	5983	1230
MiaPaca-2	789	503
Panc-1	8765	1988
